# Cycle-Based Control of Injection Moulding Process in Presence of Material Dual Sourcing Using Mass Feedback

**DOI:** 10.3390/polym16131808

**Published:** 2024-06-26

**Authors:** Rasmus Aagaard Hertz, Ole Therkelsen, Søren Kristiansen, Jesper Kjærsgaard Christensen, Frederik Agervig Hansson, Lasse Schmidt

**Affiliations:** 1 R&D Moulding, LEGO System A/S, Astvej 1, 7190 Billund, Denmark; 2 Materials Department, LEGO System A/S, Astvej 1, 7190 Billund, Denmark; 3 Moulding Analytics Center, LEGO System A/S, Astvej 1, 7190 Billund, Denmark; 4 Ngin A/S, Bautavej 1A, 8210 Aarhus, Denmark; 5 AAU Energy, Aalborg University, Pontoppidanstraede 111, 9220 Aalborg, Denmark

**Keywords:** injection moulding, part parameter control, quality control, part mass, cycle mass feedback, EWMA control, d-EWMA control, material variation, material dual sourcing

## Abstract

The low cost and precise tolerances of plastic injection moulded products are a major reason for the popularity of the manufacturing method. The tolerances are greatly influenced by the equipment, raw material and moulding process. One challenge is the raw material variation. This paper presents a production process using cycle based feedback of cycle mass, for control of part properties in the presence of material variation from dual sourcing. The part properties considered are part mass and outer dimensions. The process uses direct cycle mass feedback without additional process measurements in the proposed controller structure. The designed controller structure is tested in a multi-cavity mould while using raw materials from multiple vendors, encompassing five different grades. The results show a total decrease in part mass variance of approximately 50% and a decrease of length and width variance of approximately 40% compared to a moulding process with fixed settings.

## 1. Introduction

Injection moulding is a key process for manufacturing of plastic parts. The parts are used in a broad range of sectors such as automotive, medical and the toy industry. The demand for plastic parts is growing, and the wide use cases increase the requirement for both quality, tolerances, and competitive pricing [1,2]. The sustainable agenda in the public domain comes in addition to the increased plastic consumption in the world, resulting in greater demands for both virgin and reused plastic feedstock. Resilience in the supply chain has further put focus on the use of multiple vendors to ensure a continuous production in crisis situations. This challenges manufacturers to find methods to produce with feedstock from multiple vendors or feedstock with increased variance between batches [3,4]. Plastic parts manufactured from injection moulding are dependent on three main domains being equipment, moulding process and material. The increased variance of raw material needs to be handled to ensure the same level of quality as today.

The control of injection moulding machines has been a focus area for both industry and academia since the 1980s, where the main focus has been on closed-loop control for precision and repeatability of the machines [5]. Ensuring the precision and repeatability of injection moulding machines should in the case of constant boundary conditions result in constant part properties. However, in production environments, constant boundary conditions are unlikely to be present as, e.g., humidity, temperature and vendors of raw material changes over time [6,7].

Chen and Turng [8] described injection moulding control through a cascaded control structure with three loops; machine control, process control and part quality/parameter control. Further research within these three loops of the injection moulding process is needed to increase the controllability of the process [7]. Control strategies often rely on sensor data from the mould, even though only less than 1% of the production moulds contain sensors according to Masato et al. [9]. This requires large changes in the production setup to utilise controllers where sensors in the mould are required. The simplicity of not having sensors in the mould on the contrary makes the implementation process less demanding.

The objective of any process control system based on feedback control is to manipulate the desired states to achieve a desired objective. A method of designing a reliable controller is to gain an understanding of the system through a representative mathematical model [10]. To successfully design a process control system, three domains need to be present; the desired reference, a stable controller, and a feedback signal coming from either an observer or a sensor. A fundamental difficulty in controlling part properties of injection moulded products is that only a limited amount of product properties can be measured in-line [11]. This leads to two fundamental difficulties in closed-loop process control related to injection moulding.

- What is the desired reference/target?
- How is the feedback signal measured or observed?

**Figure 1:**
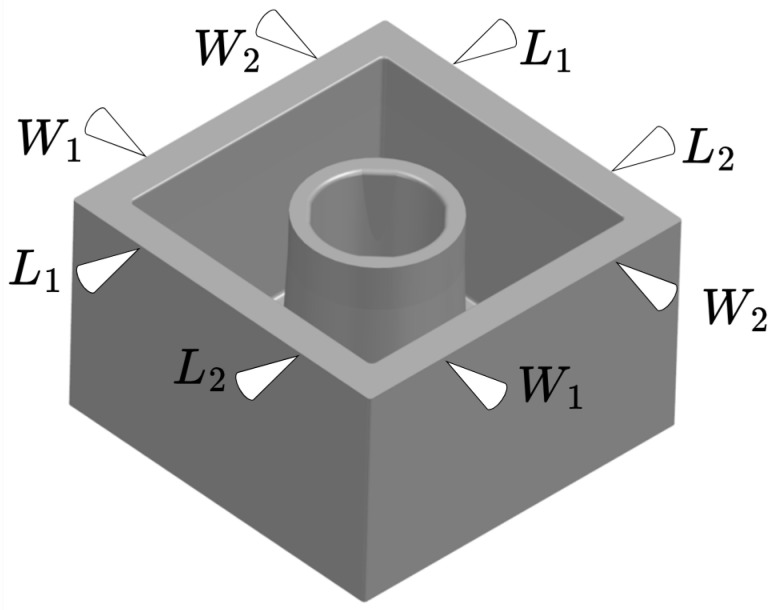
Sketch of boxlike part and measurement points.

The answer depends on in which of the three cascaded control loops is being considered. This work is based on a case study with a 16 cavity cold runner mould. The geometry of the part is primarily defined by the mould cavity; although, the material and moulding process also influence the final shape, as discussed above. Multiple part properties, such as weight, and dimensions can be important for the functionality of the produced part. Figure 1 shows a sketch of the box-shaped part used in the presented research.

The figure further shows the measurement points on the part of importance for the part functionality. The box-shaped part was selected as the box geometry is commonly used in industry. It further includes complex features such as edges, squares, and circles. The complexity of this geometry ensures that the method is applicable to various industrial applications where similar geometries are present. The properties of interest for this part are part mass (m), length $\left(L_{1},L_{2}\right)$ and width $\left(W_{1},W_{2}\right)$, where a tolerance can be set ensuring, e.g., the functionality of the produced part. When referring to length $\left(L_{1},L_{2}\right)$ and width $\left(W_{1},W_{2}\right)$ it is the distance between the two point marked by the two arrow heads with the same index as shown in Figure 1. The geometric part measurements are conducted on a coordinate measuring machine (CMM), approximately 48 h after moulding. The mass of the parts are reported either immediately after moulding or after 48 h when parts have stabilised concurrent with the CMM measurement. When single part masses are discussed or plotted it is measured after approximately 48 h, whereas when the cycle mass of the parts from a whole cycle is plotted or discussed it is on-line immediately after moulding denoted “part mass” and “cycle mass”, respectively.

### 1.1. Part Property Variance

The variance of injection moulded products can be considered from multiple perspectives and depends on the level of reference [12]. In this work, it is separated into three levels:

1. Cavity to cavity: In a multi cavity mould variance from cavity to cavity is present, which can originate from imperfect manufacturing of the mould, runner balancing, or the injection moulding process selected.
2. Cycle to cycle: The variance from cycle to cycle can originate from variation in the actuation of the injection moulding machine, changes in raw material, or long settling time of, e.g., mould temperature when starting up a new production order.
3. Variation over several cycles: Hour to hour variance could be caused by imperfect mixing of color into the raw material, imperfect mixing of regrind, change of raw material batch or vendor.

The discussion about the frequency and magnitude of the variance in relation to the part properties of interest, being part mass, length and width, is essential in order to understand the manufacturing concept proposed in this paper. In injection moulding with multi cavity cold runner moulds, there is essentially only a single input, that forces plastic into the mould, which is the injection cylinder that controls the linear movement of the screw. The process therefore has a single input and multiple outputs being a part for each cavity, rendering it impossible to control each cavity separately. In the following the frequency and magnitude of the variation is discussed. The frequency of the variance is expected to be slower for each level of perspective; however, this does not necessarily mean the magnitude of the variation have the same tendency. The cycle size of injection-moulded products is typically measured in grams or kilograms. Since the barrel often holds material for more than one cycle, the magnitude of cycle-to-cycle variance is expected to be relatively small as the batch size of raw materials is often measured in tonnes. This implies that the change in raw material can cause a larger disturbance than two consecutive cycles.

### 1.2. Previus Work

The machine manufacturer’s focus is to increase the precision of injection moulding machines, to ensure that two subsequent cycles are identical, reducing the variation from cycle to cycle [13]. The manufactures have further improved the control with smart systems, being able to alter, e.g., the switchover point if the cycle differs from the previous cycle [14]. Part mass process control schemes in injection moulding can in general be divided into two categories, observer based methods and methods based on direct mass feedback. The observer-based quality control schemes rely on either a mathematical model or data from, e.g., experiments or a combination to predict the part properties. Examples can be found in [11,15,16,17,18,19,20,21].

The observer based methods are contrary to direct feedback methods that employ a sensor to measure the desired part property directly. The sensor data may be standalone or combined with additional machine data or models. In this work, focus is on the control of part mass. Srinivasan et al. [22] describe a part mass controller based on a PI controller, weighing a single part from the moulding cycle manually and adjusting the holding pressure manually from the calculated value. The results show an improved tracking of the mean part mass with a slight increase in part mass variability when subject to process disturbances.

Havard et al. [23,24] uses the part mass together with an empirical model containing, viscosity, injection temperature, mold temperature and holding pressure to control the part mass of a fibre reinforced semi-crystalline thermoplastic. The mass is used as a corrective function of the model to avoid long term drift as a pure proportional action. The developed control strategy improves the mass stability under thermal perturbation.

Chen and Turng [25] designed a cascaded controller structure, with the outer loop utilising cycle mass feedback to correct the switchover point to maintain the cycle mass. The inner loop maintains a constant maximum mould separation, through the use of linear variable differential transformer sensors in the mould. Moulding trials shows an improved part mass consistency.

### 1.3. Objective

With the overview of the two fundamental problems in feedback control of the injection moulding process, the understanding of variation in injection moulded parts and the previous work the objective of this work is presented. The objective is to reduce the part property variance of the box-like part, produced with five different ABS materials. A delimitation is that the material being processed is unknown, making pre-measured properties such as viscosity and pressure–volume–temperature (pvT) characteristics of the five materials inapplicable. It is assumed that the mold cavities are manufactured with the necessary precision, ensuring that the relative tolerances within each part is met. Additionally, it is assumed that the variation from cycle to cycle remains within acceptable limits rendering in-cycle control unnecessary.

The approach taken to achieve the objective is outlined below to guide the reader. The hardware, moulding process and material used are first described. This is followed by a design of experiments (DoE) to show the correlation between part mass and dimension for different machine settings. This is used to establish if cycle mass can be used as feedback signal to control both part mass and dimension for the considered part. The DoE is further used to select an appropriate machine setting to manipulate. A process model based on experimental data is established from experiments with the five different ABS materials. The process model is used for selection and design of the cycle-based controller. The designed controllers are experimentally validated through moulding trials, where the moulding process is subject to a change from one material to another. The material is introduced both as a step and ramp input to the moulding process. The part properties produced with the standard process and the proposed cycle mass feedback process using the five different ABS materials are compared. The results and proposed method are discussed followed by a conclusion.

The research gap addressed is the fundamental difficulties in the feedback control of the injection moulding process, establishing the target of the controller and how the feedback signal is measured. The controller structure proposed for controlling the cycle mass is novel in relation to the injection moulding process. The findings are evaluated through experimental validation of the controller performance and the expected reduction in part property variation using five different ABS materials.

## 2. Hardware and Material

The injection moulding machine used to conduct the experiments in this work is a modified industrial injection moulding machine described in Hertz et al. [26,27]. An important sub-process to consider when adjusting machine settings on-line is the switchover process described in Hertz et al. [28]. The pressures considered in this work is hydraulic pressures, as it is the pressure directly measured. Note that injection moulding machine user interfaces often use the apparent pressure at the end of the screw, based on an intensification factor, which for this machine is approximately 1:10. The moulded parts are ejected into a container which is moved to a scale capable of measuring the cycle mass of the moulded parts. The cycle mass is digitally sent to the controller.

### 2.1. Injection Moulding Process

The injection moulding process contains several states such as, e.g., position, velocity, pressure and temperature. The moulding process considered in this work is called secondary overpoint often used in the industry [3]. It consists of a velocity-controlled fill phase and a pressure-controlled holding phase. The switchover between the two is ideally when the mould is volumetric full. Injection moulding machines contains hundreds of settings all influencing the moulding process. Some of the settings to consider are, e.g., injection velocity, barrel temperature, holding pressure, mould temperature, switchover position and dosing speed. The focus of machine vendors are to keep the machine stable, by introducing several feedback loop which partly decouples the different states. This work therefore only considers the influence of a subset of the settings on the part properties, whereas the rest are assumed constant. The selected settings are material melt temperature, injection velocity and holding pressure as these are known to influence part properties which can be explained from pvT diagrams.

### 2.2. Shrinkage of Amorphous Material

The materials utilized are five different types of Acrylonitrile Butadiene Styrene (ABS) formulations including different vendors or grades. ABS is an amorphous thermoplastic. Amorphous plastics are characterised by a homogeneous and isotropic structure, whereas, e.g., semi-crystalline materials forms structures are dependent on, e.g., the cooling rate. For amorphous materials, it is found that process states such as, e.g., cooling rates, in general do not effect the density and therefore the relation between dimensions and part mass [29]. The shrinkage of injection-moulded parts can be separated into two stages, in mould shrinkage and post mould shrinkage. Both in- and post-mould shrinkage occurs due to cooling of the material. The in-mould shrinkage can be minimised by forcing additional plastic into the cavity. This can be done from several machine settings such as, e.g., holding pressure [12]. Controlling the amount of added plastic to the cavity can be used to minimise the variance of the produced parts from cycle to cycle by adapting the moulding process dependent on the material.

The pressure and temperatures influence the shrinkage of the produced parts depending on the used material. The resulting shrinkage can in a simplified case be described through pvT diagrams [30]. The pvT diagrams describe the relation between pressure, specific volume and temperature at a given location in the mould. From the pvT diagrams, it can be seen that increasing or decreasing the temperature and pressure will influence the resulting shrinkage. This is used actively in some control strategies, where the desired trajectory in the pvT diagram is related to the moulding process and ideally generate the desired machine setting for the predicted shrinkage of the produced part, which is documented in, e.g., [7,17,30,31]. The material properties, such as viscosity, pvT, etc., vary depending on, e.g., grade, manufacturer, etc. No studies have according to Hertz et al. [3] been published where a general effective measurement of the pvT properties in-line in the injection moulding process has been proposed. For process control, this will limit the use of pvT controlled processes to known materials, and it is further required to track what material is used and when. This work considers a method where the material properties are unknown; however, the understanding of the link between process and material is used when the experiments are designed and for selection of the machine settings to study as previously discussed.

## 3. Effects of Machine Settings on Part Properties

Both the part mass and the dimensions of the part shown in Figure 1 are essential for the functionality of the part. It has previously been shown that the changes in part mass mainly is due to change in dimensions rather than density [29]. It is further previously shown that the moulding process influences the part mass and dimensions of the produced parts [16,32,33,34]. It is therefore necessary to establish if the machine settings have an effect on the relationship between part mass and dimensions. This will be explored through a DoE using one of the five ABS materials.

A DoE is set up to test the influence of machine settings on the relationship between part mass and dimension. The test is conducted as a full factorial DoE with three factors in two levels. The factors considered are holding pressure, material mass temperature and injection velocity. All other settings of the moulding machine are kept constant. The DoE are performed in two blocks, as changing material melt temperature is a slow process and is therefore used as the blocking factor, meaning all experiments are conducted at one material melt temperature before it is changed to the second level. The full factorial DoE results in eight experiments, where part mass and dimensions are measured for each cavity. Three cycles are measured for each experiment, where both part mass and dimensions are measured after 48 h.

**Figure 2:**
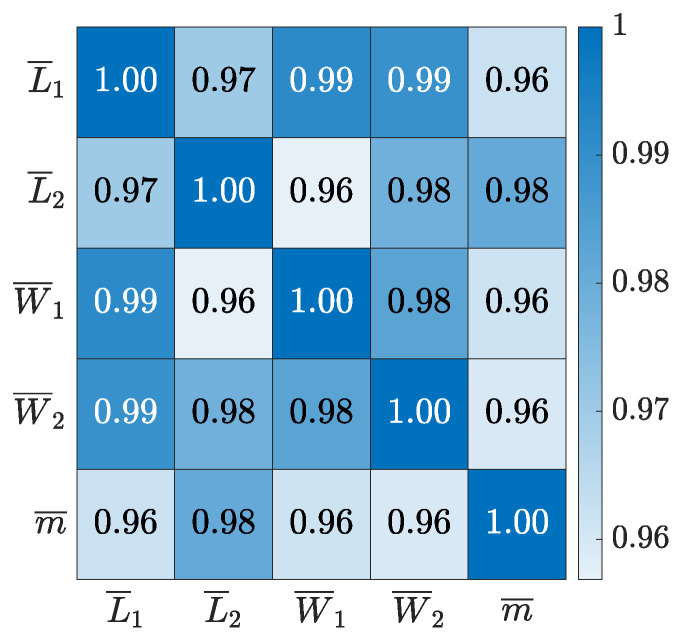
Pearson correlation between average dimensions and part mass of the box like part.

The Pearson correlation between the measurements internally are first considered. The analysis is based on the average dimensions and part mass for each experiment of the DoE. The Pearson correlation is shown as a heat plot between average length (${\bar{L}}_{1}$,${\bar{L}}_{2}$), width (${\bar{W}}_{1}$,${\bar{W}}_{2}$) and part mass ($\bar{m}$) in Figure 2.

The correlation matrix shows a significant positive correlation between the measurements, meaning that if the part gets longer it also gets wider and thicker. This makes it possible to influence the output of the part by adding additional polymer to the cavity. However, it should also be considered that it is not possible to alter the relative measurements of the dimensions internally.

**Figure 3:**
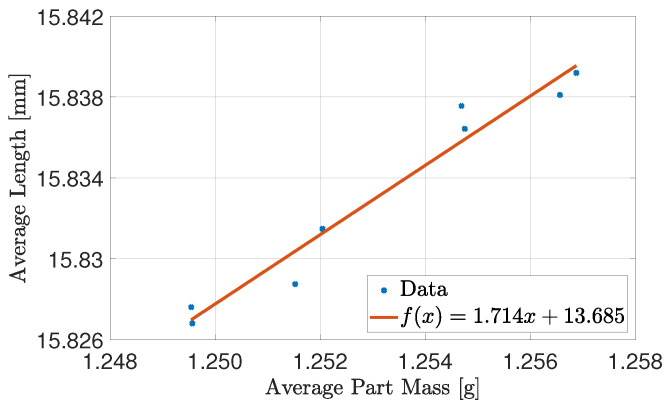
Average part length as a function of average part mass for each DoE setting.

The correlation matrix show a positive correlation between the part properties of interest. The nature of the correlation is considered for each experiment by plotting the average length by combining $L_{1}$ and $L_{2}$ as a function of average part mass as shown in Figure 3.

The experiment shows that the relation between average length of the parts and average part mass in the tested region can be modelled as a linear function with a $R^{2}\approx 0.96$. The affine function indicates that none of the tested combinations affect the relationship between part length and part mass. It will therefore be possible to control the part properties with only a single input.

### Machine Setting Selection

**Figure 4:**
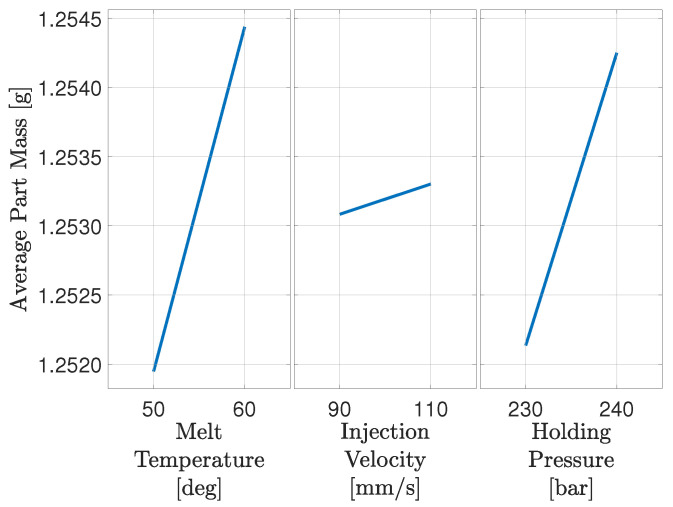
Main effect plot showing the average mass of the parts as a function of the three factors in the DoE being material melt temperature, injection velocity and holding pressure.

In the design of the closed-loop process control system, it is in general crucial to identify the state that affects the desired system behaviour. From the DoE data, it can be seen that the three factors in the described DoE all influence the part mass and dimensions. It has further been established that the interactions between machine settings do not affect the relation between dimensions and part mass. It is desired only to manipulate one setting in the closed-loop system to keep the control architecture simple. Figure 4 shows the main effect plot, between the adjusted machine settings and part mass.

The main effect plot for the DoE reveals that material melt temperature, injection velocity, and holding pressure all have an impact on the part mass. It is assumed that the injection moulding machine controller keeps the non-manipulated machine states sufficiently constant, making it unnecessary to consider the interaction with these. In this work, the machine setting chosen to manipulate is the holding pressure, due to effectiveness as it significantly impacts the part mass for the relatively low range tested. It further benefits from being able to change from cycle to cycle. The holding pressure is chosen rather than the material melt temperature, as it is possible to adjust the holding pressure from cycle to cycle, whereas the settling time of the material melt temperature is several cycles. The temperature further have an influence on the colour of the produced part resulting in an increased probability of other quality challenges. The holding pressure is chosen rather than the injection speed even though it is possible to change that from cycle to cycle. The injection speed requires a quite large range to have a comparable impact on the part mass. Additionally, it influences the shear heat resulting in a change in material melt temperature in the injection phase. This increased temperature especially in the runner system can also influence the color and is therefore kept constant. The selection of holding pressure as the manipulated machine setting is in addition consistent with the findings in Postawa and Koszkul [35].

## 4. Process Model

**Figure 5:**
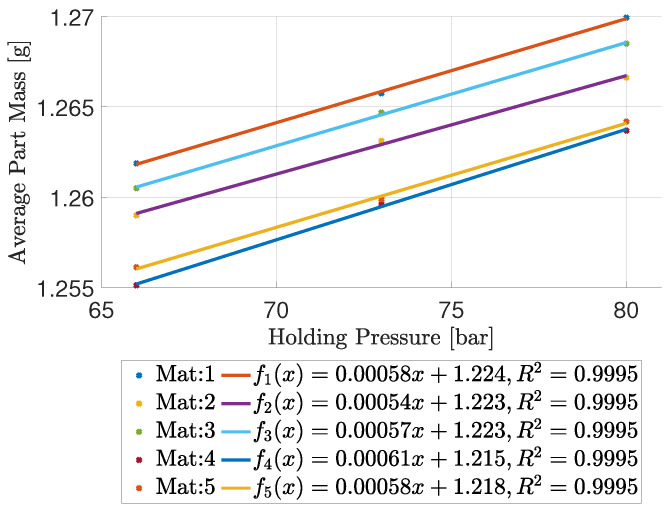
Average part mass as a function of holding pressure for five different materials.

Having established that the desired manipulated setting is the holding pressure, the model describing the connection between holding pressure and the part mass is established. The model is established based on experimental data, as it is possible to measure the necessary data while setting up the mould. The limit of experimental based models compared to theoretical models based on, e.g., physics is that it is only valid in the tested region. However, in this case, the experimental approach is preferred due to the simplicity of the test making it beneficial, compared to more complex models such as [16,20,21,34,36,37,38]. Nine experiments are conducted, where the five ABS materials are moulded at three different holding pressures. The part mass of each experiment is measured. It is chosen to include three holding pressures, as the center point is adds the possibility to observe if it is a linear relationship between part mass and holding pressure. The part mass as a function of holding pressure is shown in Figure 5.

From the figure, it is seen that the five materials have a unique relation between holding pressure and part mass locally. Additionally, it is observed that the relationship between part mass and holding pressure for all five materials locally conforms to an affine function, resembling a linear approximation as outlined in Equation (1).

(1) $$\begin{array}{c} y_{t}=\beta_{t}x_{t}+\alpha_{t}+\epsilon_{t} \end{array}$$

Here, $y_{t}$ is the output of cycle *t*, and $x_{t}$ is the input to cycle *t*. $\epsilon_{t}$ represents error as white noise, $\alpha_{t}$ is the intercept term, and $\beta_{t}$ is the gradient and process sensitivity. A linear regression has been estimated for each material, all with an $R^{2}\approx 1$. Notice that an increase in holding pressure increases the part mass, which was also concluded from Figure 4. The gradient of all tested materials has a range of approximately $7\cdot 10^{-5}$ g/bar, whereas the intercept with the y-axis has a larger range of approximately $9\cdot 10^{-3}$ g. It should be noted that the closed loop control is based on cycle mass and not part mass as plotted here, which means the values are scaled by the number of cavities when used for controller parameter selection.

## 5. Cycle-Based Cycle Mass Controller

Injection moulding is a repeatable process subject to low frequent variance from raw material changes. The cyclic nature of the process makes it suitable for cycle-to-cycle control algorithms. Cycle-to-cycle control algorithms are based on measuring an output and adjusting a machine setting for one of the next cycles.

The mathematical model, describing the relation between input being holding pressure and output being cycle mass, is in this case linear and given in Equation (1). Multiple controllers for such systems have been proposed in the literature, especially within semi-conductor manufacturing [39,40,41,42,43]. Two control strategies are considered. The first being a exponential weighted moving average (EWMA) controller and the second being a double exponential weighted moving average (d-EWMA) controller also referred to as a corrector predictor controller. The general aim of this controller type is to update the model of the process between the cycles and from this model provide a new set point for the next cycle.

### 5.1. EWMA Controller

The experimental model that describes the process in Section 4 is linear, at least locally, with a single input, indicating that the prediction model in the controller should be in the same form as the process model. The prediction model is given in Equation (2)

(2) $$\begin{array}{c} {\tilde{y}}_{t}=b_{t-1}\cdot x_{t}+a_{t-1} \end{array}$$

Here ${\tilde{y}}_{t}$ is the cycle mass of cycle *t*, and $x_{t}$ is the holding pressure setting on the injection moulding machine to cycle *t*. $a_{t-1}$ and $b_{t-1}$ are estimates of the parameters α and β, respectively, based on the cycles up to the current cycle *t*. The prediction model is used to select the holding pressure setpoint for the next run, so the cycle mass of the next cycle comes close to the target *T* satisfying $T\approx \tilde{y}$. Section 4 describes the range of the gradient β is relatively small and is for simplification purposes assumed constant meaning $b_{t-1}=constant$. The intercept term is updated for each measurement of $y_{t}$. The routine for updating *a* that takes into account the white noise in the process is based on the EWMA filter. The benefit of the EWMA filter is that the weight of the filter decays exponentially with the number of cycles. The EWMA filter is defined as Equation (3).

(3) $$\begin{array}{c} a_{t}=\sum_{i=1}^{t}\omega {\left(1-\omega \right)}^{t-1}\left(y_{i}-bx_{i}\right) \end{array}$$

where 0<ω≤1 is the EWMA weight assigned to the most recent data point. The weight value indicates how many old cycles are considered when calculating the new input. The weight ω of the EWMA filter can be tuned or calculated based on the number of old cycles considered from Equation (4).

(4) $$\begin{array}{c} \omega =\frac{2}{n+1} \end{array}$$

Here, *n* is the amount of old cycles considered. Equation (3) can be presented in recursive form as Equation (5).

(5) $$\begin{array}{c} a_{t}=\omega \left(y_{t}-bx_{t}\right)+\left(1-\omega \right)a_{t-1} \end{array}$$

Assuming the estimate $\tilde{\alpha }=a_{t}$, the control law can be stated as Equation (6).

(6) $$\begin{array}{c} x_{t}=\frac{T-a_{t-1}}{b} \end{array}$$

The designed EWMA-based controller is thereby a model-based controller. The initial value of $a_{t-1}$ is calculated based on the first cycle as Equation (7).

(7) $$\begin{array}{c} a_{0}=y_{0}-bx_{0} \end{array}$$

The stability is for a deterministic first order system where the output is measured for each cycle according to Ingolfsson and Sachs [44], asymptotically stable if Equation (8) is satisfied.

(8) $$\begin{array}{c} 0<\frac{\omega \beta }{b}<2 \end{array}$$

**Figure 6:**
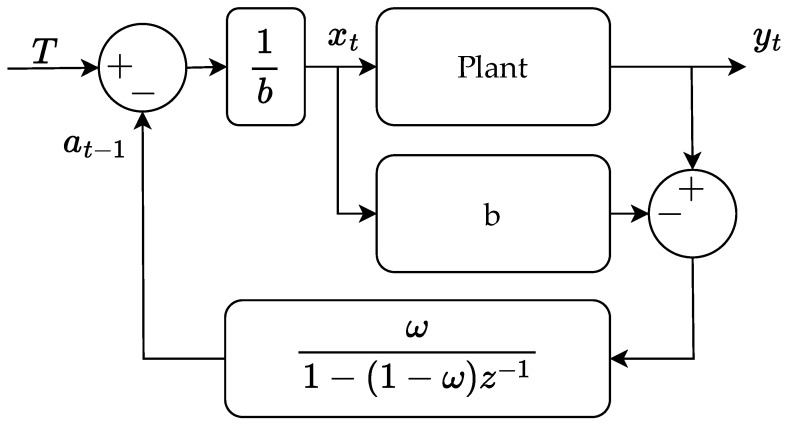
Blockdiagram of EWMA cycle to cycle controller.

This shows if β and *b* have the same sign and |b|≥β, the stability is guaranteed as 0<ω≤1. If |b|<β, the following statement must be true $|b|>\left|\frac{\omega \beta }{2}\right|$ to ensure stability. If β and *b* do not have the same sign, the process is unstable. The block diagram of the controller structure is shown in Figure 6.

### 5.2. d-EWMA Controller

The d-EWMA controller is a predictor-corrector controller, where the d-EWMA filter is used in the feedback loop. The d-EWMA controller is introduced due to the limitation of the tracking capability of the EWMA controller when severe drift per cycle is present, even if large values of ω are used. The d-EWMA controller is also based on the system described by a linear model, with a single input and constant gradient. The d-EWMA controller is stated in Equation (9).

(9) $$\begin{array}{c} x_{t}=\frac{T-a_{t}-D_{t}}{b} \end{array}$$

Here, $a_{t}$ is calculated by Equation (3), and the corrector $D_{t}$ is given in Equation (10).

(10) $$\begin{array}{c} D_{t}=\omega_{2}\left(y_{t}-bx_{t-1}-a_{t-1}\right)+\left(1+\omega_{2}\right)D_{t-1} \end{array}$$

where $0<\omega_{2}\leq 1$ is the EWMA weight assigned to the most recent data point of the system. $D_{t-1}$ is initailised according to Equation (11).

(11) $$\begin{array}{c} D_{0}=y_{0}-bx_{0}-a_{0} \end{array}$$

Castillo [45,46] has shown that if the disturbance is a; deterministic trend, random walk with drift or integrated moving average, and the output is measured for each cycle and the first order system is controlled by a d-EWMA controller, the system will be asymptotically stable if and only if the two conditions are true in Equation (12).

(12) $$\begin{array}{c} \left|1-\frac{\beta }{2b}\left(\omega +\omega_{2}\right)+\frac{1}{2}z\right|<1,\;\;\;\;\;\left|1-\frac{\beta }{2b}\left(\omega +\omega_{2}\right)-\frac{1}{2}z\right|<1 \end{array}$$

where

(13) $$\begin{array}{c} z=\sqrt{{\left(\frac{\beta }{b}\right)}^{2}{\left(\omega +\omega_{2}\right)}^{2}-4\omega \omega_{2}\frac{\beta }{b}} \end{array}$$

**Figure 7:**
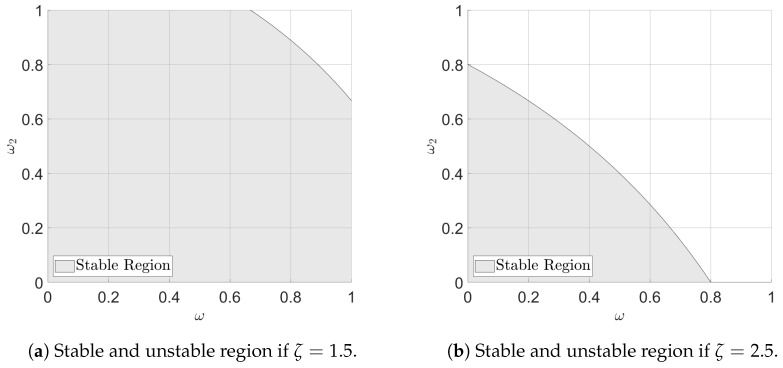
d-EWMA stability considerations for ζ=1.51 and ζ=2.5. Indicating the modelled gain is overestimated compared to the system gain.

From Equations (12) and (13), Castillo [45,46] shows that by defining $\zeta =\frac{\beta }{b}$ the stability can be evaluated. The system is unstable if −inf<ζ<0. For 0<ω<1 and $0<\omega_{2}<1$ and for ζ≤1, the system is stable. If ζ>1, a graphical approach is used to indicate if the system is stable. The stable region is shown for ζ=1.5 and ζ=2.5 in Figure 7.

**Figure 8:**
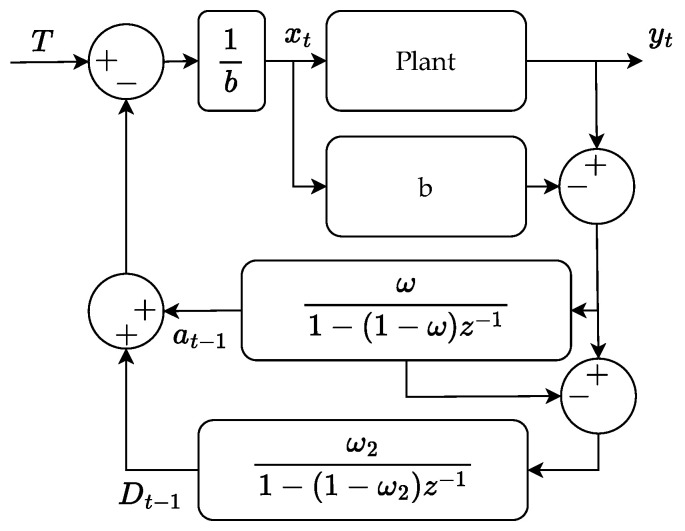
Block diagram of the d-EWMA cycle to cycle controller.

The grey area yields a stable system for the given ζ. In Figure 5, it is shown that all materials show a similar gradient, and it is expected that ζ≈1 meaning the values of ω and $\omega_{2}$ can be chosen freely between 0 and 1 ensuring a stable system. The block diagram of the controller structure is shown in Figure 8. Sudden shifts in the process, coming from, e.g., step changes in raw materials in injection moulding can result in relatively slow settling times, as the two EWMA filters consider multiple previous cycles run in a different materials. Utilising gain scheduling can minimize the influence of non-representative old cycles in situations where sudden jumps occur. The gain still has to satisfy the stability criterion for the process to be stable. In the experiments shown, the gain is adapted if the error is above a threshold and kept at the new value for seven cycles before returning to the baseline. Having the controllers described, the performance of the controller is considered when changing from one plastic feedstock to another, both as a step change and as a change emulating a ramp where the two materials are mixed slowly.

## 6. Controller Evaluation

**Figure 9:**
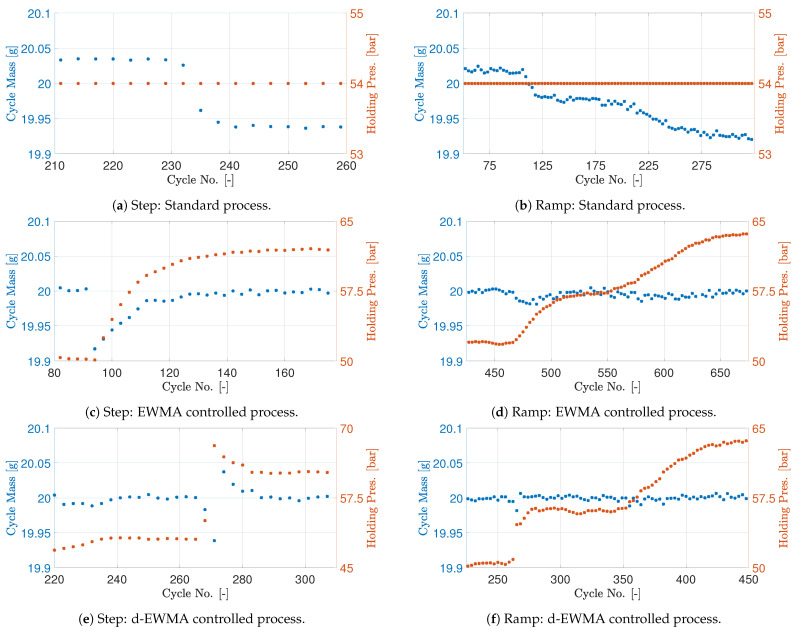
Cycle mass and holding pressure reference of standard, EWMA and d-EWMA process, subject to change of raw material. The changes in raw material presented are sudden change (step) and slow change (ramp) from one material to another material.

The designed controllers are tested separately and compared with a standard production setup without cycle mass feedback. Two scenarios are tested, one where the raw material is changed instantly emulating a step change and one where the two raw materials are mixed slowly emulating a ramp change. The results are shown in Figure 9. When the cycle mass controller is active, the target mass is 20 g. Due to speed limitations of the weighing setup, only every third cycle is measured. The measurement frequency is constant, and it is assumed the change between measurements is insignificant.

The experiments emulating a step change in material is first considered and is shown in Figure 9a,c,e. As there is material for multiple cycles in the screw, the exact moment where the second material is introduced is unknown, and it is therefore not marked in the figures. However, the sudden change in the cycle mass indicates when the new material is producing. From Figure 9a, the cycle mass changes over a few cycles from approximately 20.05 g to 19.95 g, while the machine maintains a constant holding pressure setting. Figure 9c shows that the EWMA controller is capable of rejecting the disturbance to the process when the second material is introduced. The cycle mass is returned to the desired value within 25 cycles. The exponential increase in holding pressure setpoint is present as expected due to the controller design. The performance of the d-EWMA controller with gain scheduling is shown in Figure 9e and is also capable of returning the cycle mass to the target of 20 g within 10 cycles. The overall error is furthermore smaller at all measurement instances compared to the EWMA controller. Both controllers are stable and capable of adapting the pressure to the reference of 20 g.

The experiments emulating the ramp change in material is shown in Figure 9b,d,f. The new material is added to the hopper to slowly blend in and change from one material to the other, and the change in cycle mass indicates when the second material is introduced to the process. The standard process is shown in Figure 9b and shows the same change in cycle mass just slower compared to the step material change. The absolute value of cycle mass before and after the material change vary slightly. The response of the process with the EWMA controller is shown in Figure 9d. The controller maintains the cycle mass at a constant level; however, right when the material starts to change at cycle number 475, the controller shows a lack of tracking capability and a small drift in the cycle mass is present. The gain scheduled d-EWMA controller response shown in Figure 9f, however, shows tracking capability in the full conversion from one material to the other. The variance is smaller compared to the EWMA-controlled experiment, as expected. The variation between the cycles in the tests with ramped change in material is a little larger compared with the step test. This is expected to come from the ever-changing percentage of the two materials in each cycle. In general, some variance in the measurement is expected, as in cycle variations of, e.g., screw valve closure are not accounted for in cycle-based control. Lastly, it is noted that the holding pressure machine setpoints are different when the step response in Figure 9c,e is compared with the ramp response in Figure 9d,f. The reason for this can be small changes in the raw material, drift in the cycle mass measurement device, drift in machine capability, etc., and will not be considered in further detail here.

## 7. Part Property Evaluation

The designed cycle-based controllers of the injection moulding process have proven to be stable and have proven to maintain the cycle mass at a desired cycle mass reference. To test how effective the controller is in relation to the desired part properties defined in Section 1.1, being length, width and part mass, an additional experiment is conducted. To show the impact of the cycle-based control approach, the experiment is conducted with the process using cycle mass feedback and a standard process using fixed machine settings. The considered case is for steady state, where both EWMA and d-EWMA show similar results. In this case, it is chosen to use the EWMA controller.

**Figure 10:**
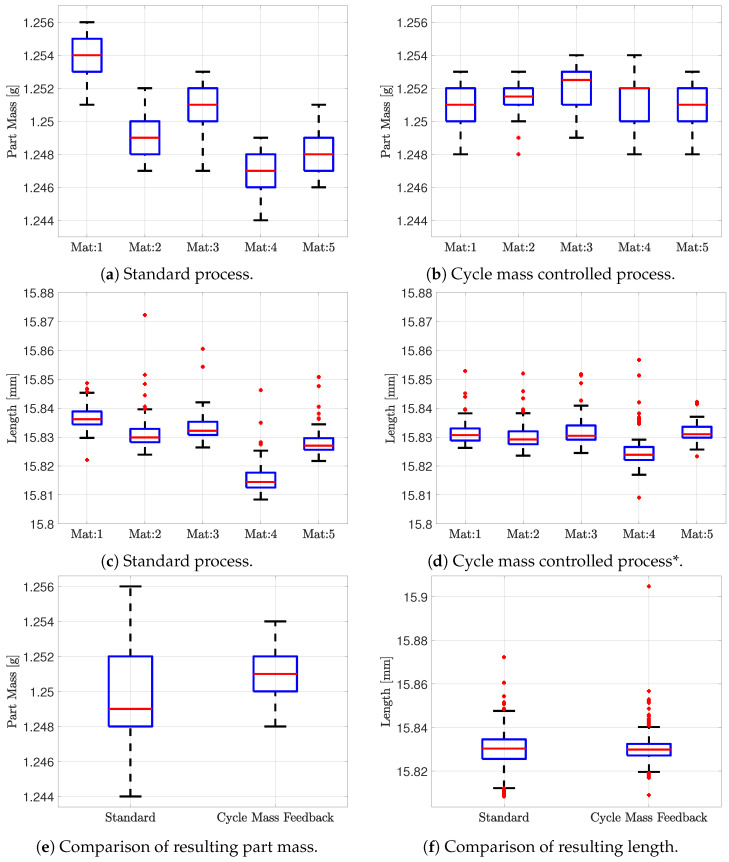
Comparison of the part properties for the standard process and cycle mass controlled process. “*” One outlier removed from Mat:4, for completeness it is shown in Figure 10f.

Five materials are tested with fixed machine settings and again with the designed EWMA cycle mass controller. Each material is used until a steady state is reached, which occurs after approximately 30 min, after which samples are taken. The samples are then stored for at least 48 h before being measured. The produced parts from three cycles are weighed, and three cycles are used for measuring dimensions for each experiment. As the test mould have 16 cavities and 10 experiments are conducted, the result is based on 480 data points for the part mass and for each length $L_{1}$ and $L_{2}$. As the length is supposed to be the same, both the dataset for $L_{1}$ and $L_{2}$ are merged, which results in 960 data points for the length of the part. The two moulding processes are compared using box plots, as it is possible to represent the distribution as well as any outliers. The measured data are shown in Figure 10.

The figure contains both length and part mass of the measured samples, together with a comparison of the overall dimensional and part mass distribution with and without cycle mass feedback. From Figure 10a,b, it is noticed that the distribution for each material is the same with and without cycle mass feedback, meaning the controller does not add or decrease cycle to cycle variance which is aligned with the objective of the control strategy. Considering the overall part mass distribution across all five materials shown in Figure 10e, an improvement of 50% is shown when using the designed cycle mass feedback control structure compared to the standard process.

The dimensional measurement shows a larger amount of outliers compared to the part mass measurement, complicating the comparison. The outliers can originate from several factors in and after the moulding process and will not be discussed in further details here. The amount of outliers are similar with and without control and is therefore neglected in the comparison. It is further noticed that the variance within each material is similar both with and without control of the cycle mass results as shown in Figure 10c,d. This is also expected due to the strong correlation shown in Figure 2. The overall reduction in length variance is shown in Figure 10f and is approximately 42%, excluding outliers. Comparing the median length of the five materials of the process with and without cycle mass feedback shows a variation reduction of approximately 70%. The results for the width are not plotted; however, they are similar to the length with a decrease in width variance of approximately 38%, excluding outliers, when using the designed cycle mass feedback control structure compared to the standard process.

## 8. Discussion

The work presented in this paper is an approach for simple part property control of parts moulded with ABS that can be adapted to any machine with access to the machine controller. The approach does not rely on material data or sensors within the mould, making it suitable for retrofitting on existing machines. The controlled property is the cycle mass, which for the tested case correlates well with dimensions. The proposed method ensures a variation reduction of part length, width and part mass in the presence of different materials using cycle mass feedback. In the presented case, the target cycle mass is the same. An alternative consideration is to run with different set points for each material, this could improve the dimensional accuracy even further. This would however be based on additional input to the controller as it is expected previous knowledge about the material or experiments investigating what the desired target cycle mass should be for different materials is needed. The additional benefit of part cycle mass feedback is the possibility of 100% cycle mass control, which means it is possible to set limits for the acceptable cycle mass, being able to scrap cycles when the cycle mass is not within limits.

The need for part property feedback control within injection moulding is increasing due to the requirement for sustainable material usage and the general focus on a reduction in the environmental impact. The direct measurement on the desired product is a leaver for this as the simplicity in this setup only requires tuning of up to three parameters, which can be performed when the mould is qualified. This increases the robustness of the production setup and makes it less dependent on knowledge of the incoming plastic feedstock. Additionally, it makes the production setup less sensitive for calibration as long as the cycle mass feedback is of a high quality, the variance from machine to machine is suppressed.

Weighing of parts in a production environment is not a simple task due to the level of background noise from surrounding equipment in the measurement. The quality of the proposed control architecture is dependent on the quality of the feedback signal. This have to be considered when choosing cycle mass set point, rejection limits and how the cycle mass is determined.

## 9. Conclusions

This paper presents an architecture for direct cycle mass control in an injection moulding process, where EWMA and d-EWMA cycle-based controllers are tested. Both controllers are shown to be capable of tracking the desired cycle mass, in presence of both slow and sudden shifts in raw material, by adaption of the moulding process. The quality criteria for the selected part have been length, width, and part mass. Experimental results running with five different raw materials have shown that the controller is capable of reducing the overall part mass difference with approximately 50% and decreasing the dimensional variance by approximately 40% excluding outliers. The proposed cycle-based architecture using cycle mass feedback, can be implemented without modifications to injection moulding machine or mould by adding, e.g., controller and weight to the production cell. The benefits are discussed both when raw materials vary in a traditional injection moulding setup, but also looking into the future where sustainable feedstock with an increased amount of variation is to be adopted.

## Data Availability

The datasets presented in this work are not available because of commercial considerations. Requests to access the data should be directed to the corresponding author.
